# Evidence for GABA-Induced Systemic GABA Accumulation in *Arabidopsis* upon Wounding

**DOI:** 10.3389/fpls.2017.00388

**Published:** 2017-03-22

**Authors:** Sandra S. Scholz, Jaiana Malabarba, Michael Reichelt, Monika Heyer, Frank Ludewig, Axel Mithöfer

**Affiliations:** ^1^Institute of General Botany and Plant Physiology, Friedrich Schiller UniversityJena, Germany; ^2^Department of Bioorganic Chemistry, Max Planck Institute for Chemical EcologyJena, Germany; ^3^Graduate Program in Cell and Molecular Biology, Biotechnology Center, Federal University of Rio Grande do SulPorto Alegre, Brazil; ^4^Department of Biochemistry, Max Planck Institute for Chemical EcologyJena, Germany; ^5^Division of Biochemistry, Department of Biology, University of Erlangen-NurembergErlangen, Germany

**Keywords:** γ-aminobutyric acid, herbivory, *Spodoptera littoralis*, MecWorm, calcium, plant defense, systemic signaling

## Abstract

The non-proteinogenic amino acid γ-aminobutyric acid (GABA) is present in all plant species analyzed so far. Its synthesis is stimulated by either acidic conditions occurring after tissue disruption or higher cytosolic calcium level. In mammals, GABA acts as inhibitory neurotransmitter but its function in plants is still not well understood. Besides its involvement in abiotic stress resistance, GABA has a role in the jasmonate-independent defense against invertebrate pests. While the biochemical basis for GABA accumulation in wounded leaves is obvious, the underlying mechanisms for wounding-induced GABA accumulation in systemic leaves remained unclear. Here, the *Arabidopsis thaliana* knock-out mutant lines *pop2-5*, unable to degrade GABA, and *tpc1-2*, lacking a wounding-induced systemic cytosolic calcium elevation, were employed for a comprehensive investigation of systemic GABA accumulation. A wounding-induced systemic GABA accumulation was detected in *tpc1-2* plants demonstrating that an increased calcium level was not involved. Similarly, after both mechanical wounding and *Spodoptera littoralis* feeding, GABA accumulation in *pop2-5* plants was significantly higher in local and systemic leaves, compared to wild-type plants. Consequently, larvae feeding on these GABA-enriched mutant plants grew significantly less. Upon exogenous application of a D_2_-labeled GABA to wounded leaves of *pop2-5* plants, its uptake but no translocation to unwounded leaves was detected. In contrast, an accumulation of endogenous GABA was observed in vascular connected systemic leaves. These results suggest that the systemic accumulation of GABA upon wounding does not depend on the translocation of GABA or on an increase in cytosolic calcium.

## Introduction

The four carbon non-proteinogenic amino acid γ-aminobutyric acid (GABA) is present in a multitude of organisms and was also detected in all plant species analyzed so far (Shelp et al., 2009). In contrast to mammals – where GABA acts as inhibitory neurotransmitter by regulating ion channels – its function in plants is still not completely understood. It seems that the role of GABA in plants is quite diverse. Several studies indicate that GABA is not only involved in regulating metabolic pathways like the Krebs cycle, it additionally acts as a signaling molecule in plant growth and development (Bouche and Fromm, 2004; Fait et al., 2008). For example, it was reported that a tightly regulated GABA level is required for optimal root and pollen tube growth (Palanivelu et al., 2003; Mirabella et al., 2008; Renault et al., 2011). Additionally, it was shown that various abiotic as well as biotic stress stimuli induce an elevation of the GABA level in plant tissue (Bown and Shelp, 1997; Kinnersley and Turano, 2000). For example, it was observed that salt and cold stress or tissue damage of soybean (*Glycine max*) leaves leads to rapid accumulation of GABA up to 25-fold (Wallace et al., 1984; Ramputh and Bown, 1996). *Arabidopsis thaliana* plants producing a lower constitutive level of GABA showed a higher susceptibility to drought stress due to a stomata closure defect which could be rescued by increasing the internal GABA level (Mekonnen et al., 2016). Also, external application of GABA to *Oryza sativa* seedlings and *Piper nigrum* plants could enhance the individuals’ performance under heat and drought stress conditions, respectively (Nayyar et al., 2014; Vijayakumari and Puthur, 2016).

Previous studies suggest that GABA acts as a player in plant defense against insect herbivores, although the exact impact of an elevated GABA concentration on the invertebrates is not clear (Ramputh and Bown, 1996; Bown et al., 2006; Scholz et al., 2015; Bown and Shelp, 2016). On the one hand, insects feeding on high concentration of GABA show a reduced performance. Rearing lepidopteran *Choristoneura rosaceana* cv Harris larvae on artificial diet supplemented with 1.6–2.6 μmol GABA (g fresh weight)^-1^ reduced growth and survival of the insects and delayed their development (Ramputh and Bown, 1996). Similar, *Spodoptera littoralis* larvae fed with a GABA concentration up to 1 μmol (g food)^-1^ gained less weight compared with the control group (Scholz et al., 2015). On the other hand, attacked plants had an elevated level of GABA in plant tissue in response to herbivore contact. The movement of crawling insects on the leaf surfaces of *Nicotiana tabacum* and *Glycine max* plants was sufficient to stimulate the local GABA level by five-fold after 5–10 min (Bown et al., 2002). In *Arabidopsis thaliana* Col-0 plants, feeding of *S. littoralis* larvae for 3 h increased the local GABA concentration by two-fold (Scholz et al., 2015). Interestingly, continuous mechanical wounding with the robotic caterpillar MecWorm (Mithöfer et al., 2005) resulted in an up to 10-fold elevation of the local endogenous GABA level (Scholz et al., 2015).

In *Arabidopsis*, GABA is mainly produced from L-glutamate; the decarboxylation reaction is catalyzed by glutamate decarboxylases (GADs), which are encoded by five genes. GAD1 and GAD2 are the most abundantly expressed members of this family (Shelp et al., 2012; Scholz et al., 2015). It was shown that GAD activity is induced by two different mechanisms: (i) in intact plant tissue and neutral pH GAD activity is stimulated in a Ca^2+^-dependent manner by binding of calmodulin to the CAM-binding site (Snedden et al., 1995); (ii) after wounding of plant cells the vacuolar content is released and the cytosol is acidified which leads to a Ca^2+^-independent activation of GADs (Carroll et al., 1994). The degradation of GABA is carried out in the mitochondrial matrix by a GABA-transaminase (GABA-T/POP2). In *Arabidopsis*, GABA-T is encoded by only one gene whose knock-out results in constitutive elevated level of GABA in the *pop2-5* plant (Palanivelu et al., 2003).

In recent studies, the focus in plant wounding-induced stress signaling shifted from only analyzing the locally treated leaf to considering the whole plant response including the systemic leaves (Farmer et al., 2013; Mousavi et al., 2013; Kiep et al., 2015). When the plant was wounded or attacked by a herbivore on one leaf, a spike in cytosolic calcium, [Ca^2+^]_cyt_, was generated locally as well as in adjacent leaves with vascular connections to the treated leaf. In *Arabidopsis thaliana* plants, wounding leaf 8 including its midrib, caused a [Ca^2+^]_cyt_ elevation in vascular connected leaves 5, 8, and 13 (Dengler, 2006; Kiep et al., 2015). The response in the local leaf was immediate whereas the response in the systemic leaves showed a delay of 1–2 min. A similar response pattern was observed for plants fed by *S. littoralis* larvae (Kiep et al., 2015). Strikingly, the [Ca^2+^]_cyt_ elevation in adjacent leaves after wounding is dependent on the presence of the Ca^2+^-permeable vacuolar channel TWO PORE CHANNEL 1 (TPC1-2) (Kiep et al., 2015). The systemic signaling is quite complex and the elevation of [Ca^2+^]_cyt_ was accompanied by e.g., an increase in ROS and electrical signals moving to systemic tissues of the plant (Gilroy et al., 2016). Additionally, it was observed that a precursor of the defense-related phytohormone jasmonic acid (JA) was able to travel from the wounded local tissue to unwounded systemic leaves when applied exogenously (Jimenez-Aleman et al., 2015). Similarly, a systemic accumulation of GABA in unwounded adjacent leaves was detected after mechanical wounding of one single defined leaf in *Arabidopsis thaliana* (Scholz et al., 2015).

Thus, the open question is how insect feeding or wounding initiates the systemic accumulation of GABA in the plant. Two different mechanisms are conceivable. First, the observed systemic cytosolic calcium elevation could trigger the production of GABA in the systemic leaves by interaction of GADs with calmodulin. Second, no signaling is involved but GABA itself can travel from the treated leaf through the vascular system to the systemic leaves. Here, we addressed and challenged these hypotheses. Therefore, we used an *Arabidopsis tpc1-2* mutant lacking a systemic cytosolic calcium signal after wounding to study the impact of calcium. In addition, we characterized the *pop2-5* mutant upon wounding and herbivore attack and, due to its inability to degrade GABA, employed this mutant line in transport studies with D_2_-labeled GABA.

## Materials and Methods

### Plant Growth and Treatment

Four to 5-week-old plants grown in 10 cm round pots were used for all experiments. *Arabidopsis thaliana* Columbia-0 wild-type and mutant plants (*tpc1-2* and *pop2-5*) were kept at short day conditions after stratification for 2 days at 4°C. The growth chamber was adjusted to 50–60% humidity and 21°C with a 10-h-light/14-h-dark photoperiod using FLUORA^®^ L 36W/77 bulbs (OSRAM, Garching, Germany) with a light intensity of 100 μmol m^2^ s^-1^. Seeds of *tpc1-2* were kindly provided by Prof. Edgar Peiter (University Halle) and *pop2-5* seeds (GK_157D10) were purchased from GABI-Kat directly (Kleinboelting et al., 2012). To ensure the same starting conditions, all plants used for one assay were sown and germinated on the same day and were kept in the same growth chamber. For experiments investigating the systemic response and translocation of metabolites the leaves of each plant were counted according to their age (Dengler, 2006; Farmer et al., 2013; Kiep et al., 2015).

MecWorm treatment was used for mechanical wounding of the plant with punches every 5 s, totaling 12 punches per minute on treated leaf 8 (Mithöfer et al., 2005; Scholz et al., 2015). To investigate the systemic response upon treatment of leaf 8, the local and systemic leaves 5, 8, 9, 11, and/or 13 were analyzed. Untreated plants, kept exactly as the treated plants, were used as controls. The duration of the MecWorm treatment used is indicated in the respective figures. To study the wounding-induced systemic translocation of GABA in the plant, a double-deuterated GABA (Sigma–Aldrich, Munich, Germany) was used and applied to *pop2-5* mutant plants. The *pop2-5* mutant was used for this assay since it cannot degrade GABA due to the knock-out of the *GABA-T* gene (Palanivelu et al., 2003). According to (Jimenez-Aleman et al., 2015), 20 μl of 50 μM D_2_-GABA or water was applied to local wounds on leaf 8, which were generated with a pattern wheel (PRYM_610940, Prym, Stolberg, Germany) without wounding the midrib. On each side of the midrib, the leaf was wounded with six vertical motions. D_2_-GABA and water-treated plants were kept for 1.5 and 3 h with a cover to prevent evaporation. Samples of leaf 8 and chosen systemic leaves were harvested after the indicated time points. To avoid additional accumulation of GABA due to the cutting and harvesting process, all samples were harvested in less than 1 min and directly stored in liquid nitrogen. For all plants the leaves were harvested from oldest to youngest leaf (starting with leaf 5). All samples were kept at -80°C till further analysis.

### Insect Material and Feeding Assays

Larvae of the generalist herbivore *Spodoptera littoralis* were hatched and reared on artificial diet (Bergomaz and Boppre, 1986) at 23–25°C with 10-h-light/14-h-dark cycles. For the 1 week long-term feeding assay, three larvae of first instar were placed on every wild-type and mutant plant. To achieve similar starting conditions, all larvae determined for one plant genotype were pooled and weighed prior to the experiment. The minimal starting weight of 30 larvae was set to 60 mg to ensure a survival of all larvae. After 1 week the weight of every larva was recorded separately. For the short-term feeding assay third instar larvae were used after they were kept overnight without food. This treatment ensures an immediate start of feeding after placement on the plant. The locally fed leaves were collected after the indicated time points and kept at -80°C till further analysis.

### Extraction and Quantification of GABA

Two hundred and fifty milligram of fresh leaves were frozen in liquid nitrogen and weighed to enable a GABA determination per g fresh weight. If multiple leaves of a plant were collected, then leaves were cut according to their age starting with the oldest (leaf 5). The leaf material was homogenized in a Geno/Grinder^®^ 2010 (Spex Sample Prep, Stanmore, UK) equipped with aluminum racks. The racks were cooled in liquid nitrogen prior to usage to prevent a thawing of leaf material during the whole homogenization process. The amino acids (including GABA) were extracted twice with a total of 2 ml of methanol on ice. Supernatants were combined and dried using a Concentrator plus (Eppendorf, Hamburg, Germany) and re-suspended in 500 μl of methanol. The extract was diluted 1:20 (v/v) with water containing the internal standard. The algal amino acid mix ^13^C, ^15^N (Isotec, Miamisburg, OH, USA) was used as internal standard at a concentration of 10 μg ml^-1^ in all samples. The concentration of unlabeled and D_2_-GABA (M + 2) was analyzed by LC-MS/MS according to previous studies (Scholz et al., 2015). An API 5000 tandem mass spectrometer (Applied Biosystems, Darmstadt, Germany) was operated in positive ionization mode with multiple reaction monitoring (MRM) to monitor analyte parent ion → product ion: GABA (*m/z* 104.1 →87.1; DP 51, CE 17); this MRM is specific for GABA and does not detect any α-aminobutyric acid, D_2_-GABA (*m/z* 106.1 →89.1+ *m/z* 106.1 →88.1; DP 51, CE 17).

### Extraction and Quantification of Phytohormones

Like the quantification of GABA, 250 mg of leaf material was used for phytohormone analysis. The extraction procedure and determination of JA and JA-Ile was carried out on ice as described before (Vadassery et al., 2012) with small changes. In this study, a different mixture of labeled jasmonates was used as internal standard. Instead of 15 ng of JA-[^13^C6]-Ile conjugate used in the previous study, 60 ng of D_6_-JA-Ile (HPC Standards GmbH, Cunnersdorf, Germany) was used. Additionally, the 60 ng of 9,10-D_2_-9,10-dihydrojasmonic acid was replaced by 60 ng of D_6_-JA (HPC Standards GmbH, Cunnersdorf, Germany). Since it was observed that both the D_6_-labeled JA and JA-Ile contained 40 % of the corresponding D_5_-labeled compounds (which were not included in the analysis method), the obtained results were divided by 1.7 to exclude this inaccuracy.

### Statistics

All plants of different mutant lines used for one experiment were grown in the same growth chamber. Independent experiments were treated as a completely randomized design. Experiments were repeated three times to ensure reproducibility and 5–10 plants were used in each treatment for each experiment time point. Data of all independent experiments were pooled and analyzed. For comparison of two groups, the Student’s *t*-test or Mann Whitney *U*-test was applied. For statistical analyses of multiple groups, one-way analysis of variance (one-way ANOVA) or two-way analysis of variance (two-way ANOVA) were used as indicated in the figure legends. Different letters indicate significant differences between treatments. GraphPad Prism 6 and Origin Pro were used for data analysis and graph composition.

## Results and Discussion

### GABA Accumulates in Systemic Untreated Leaves in the Calcium-Channel Mutant *tpc1-2*

**FIGURE 1:**
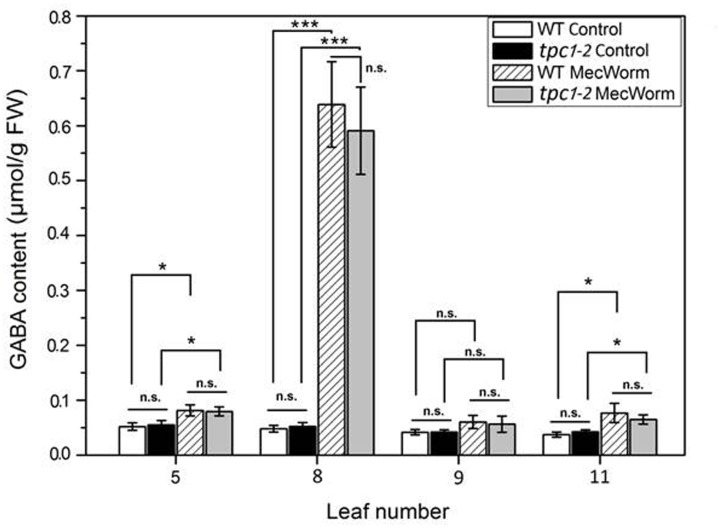
**Accumulation of GABA in individual *Arabidopsis* leaves of wild-type (WT) and *tpc1-2* plants after MecWorm treatment.** Mean [±SE, *n* = 6 (control), 8 (MecWorm)] levels of GABA were determined in individual leaves of untreated control plants and plants after 1.5 h treatment with MecWorm. In treated plants, leaf 8 was subjected to mechanical damage and systemic leaves 5, 9, and 11, and treated leaf 8 were analyzed for GABA level. Significant differences between the GABA level in the same leaf of the control and treated plant were analyzed by *t*-test (for each leaf separately, *p* < 0.05, Mann–Whitney *U*-test), ^∗^*P* ≤ 0.05; ^∗∗∗^*P* ≤ 0.001.

To examine if the production of GABA in untreated systemic leaves is induced in a Ca^2+^/calmodulin-dependent manner (Snedden et al., 1995), the systemic GABA accumulation was analyzed after MecWorm treatment in the *tpc1-2* mutant. Recently, it was observed, that the *tpc1-2* mutant did not show a systemic [Ca^2+^]_cyt_ elevation after mechanical wounding (Kiep et al., 2015). Thus, *tpc1-2* is a good model to study whether or not the systemic GABA accumulation depends on the systemic calcium signal. As expected, after 1.5 h of MecWorm treatment on WT leaf 8, this leaf showed the highest GABA accumulation. However, a significant increase in GABA concentration was also observed in systemic leaves 5 and 11 (**Figure 1**). With 0.05 μmol g FW^-1^ the *tpc1-2* plants showed the same basic GABA level in control plants as the WT and also a comparable increase in systemic leaves 5 and 11 (**Figure 1**). This indicates that the insect-like wounding-induced systemic [Ca^2+^]_cyt_ elevation has no or limited influence on the amount of GABA produced in systemic leaves. For soybean GAD it was observed that an increase in [Ca^2+^]_cyt_ up to 7–11 μM is necessary to decrease the enzyme’s Km value about 55% (Snedden et al., 1995). The determined stress-induced [Ca^2+^]_cyt_ elevations in *Arabidopsis* showed an increase to a maximum of 0.6–2 μM (Knight et al., 1996, 1997; Whalley and Knight, 2013). Hence, the wounding-induced systemic [Ca^2+^]_cyt_ elevation appears to be insufficient to account for the increasing GAD activity observed in the present study.

### The GABA Accumulating *pop2-5* Mutant Responds Efficiently to Mechanical Wounding and *Spodoptera littoralis* Feeding

**FIGURE 2:**
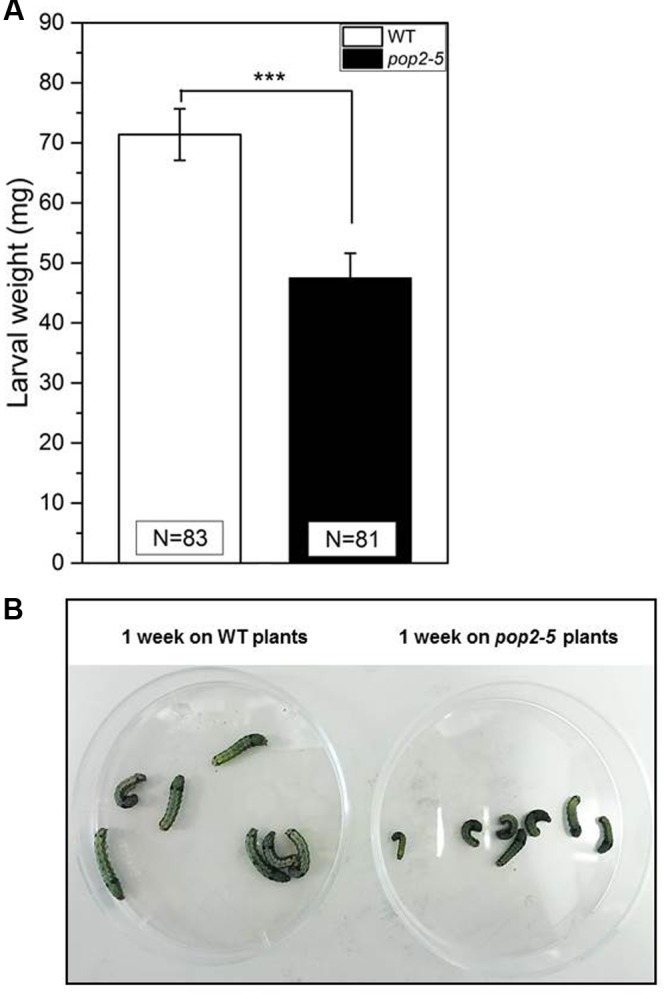
**Feeding assay of *Spodoptera littoralis* larvae on *Arabidopsis* WT and *pop2-5* plants.** *S. littoralis* first instar larvae were pre-weighed and three larvae were placed on each plant. The larval weight (mean ± SE) was measured after 7 days of feeding. **(A)** Final weight of larvae after feeding for 1 week on WT (white) and *pop2-5* (black) plants. The total number of larvae weighed (N) is indicated in the bars. **(B)** Larval macroscopic view after 1 week of feeding on the indicated mutants. Experiments were repeated three times independently. Significant differences between WT and GABA mutant plants after feeding were analyzed by Student’s *t*-test (^∗∗∗^*P* ≤ 0.001).

Due to the results obtained with *tpc1-2* plants, the possibility of direct GABA transport to systemic leaves was investigated. Therefore, we employed the GABA-transaminase knock-out mutant line, *pop2-*5. This mutant has not been used and characterized in the context of herbivory. Previous studies observed that increased concentrations of GABA can affect insect’s development (Ramputh and Bown, 1996; MacGregor et al., 2003; Bown et al., 2006; Scholz et al., 2015). We used the *pop2-5* mutant to further evaluate the effect of high endogenous GABA concentration *in planta* on the herbivorous larvae of *Spodoptera littoralis* in a feeding assay (**Figure 2**). Our results demonstrate that the larvae, feeding 1 week on *pop2-5* plants, gained significantly less weight than the larvae feeding on WT plants (**Figure 2A**). The difference could also be observed by macroscopic inspection (**Figure 2B**). In previous experiments with GABA-supplemented artificial diet it was shown, that larvae of *C. rosaceana* and *S. littoralis* feeding on high levels of GABA showed decreased survival, delayed development and decreased gain of weight, respectively (Ramputh and Bown, 1996; Scholz et al., 2015). This indicates that the constitutive GABA accumulation in *pop2-*5 plants seems to contribute to the enhanced resistance against *S. littoralis* larvae attack while the exact effect of GABA on the insects is not known. Enhanced GABA levels in the insect might lead to permanent activation of GABA-activated Cl^-^-channels resulting in hypertension or paralysis (Sattelle, 1990; Bown et al., 2006).

**FIGURE 3:**
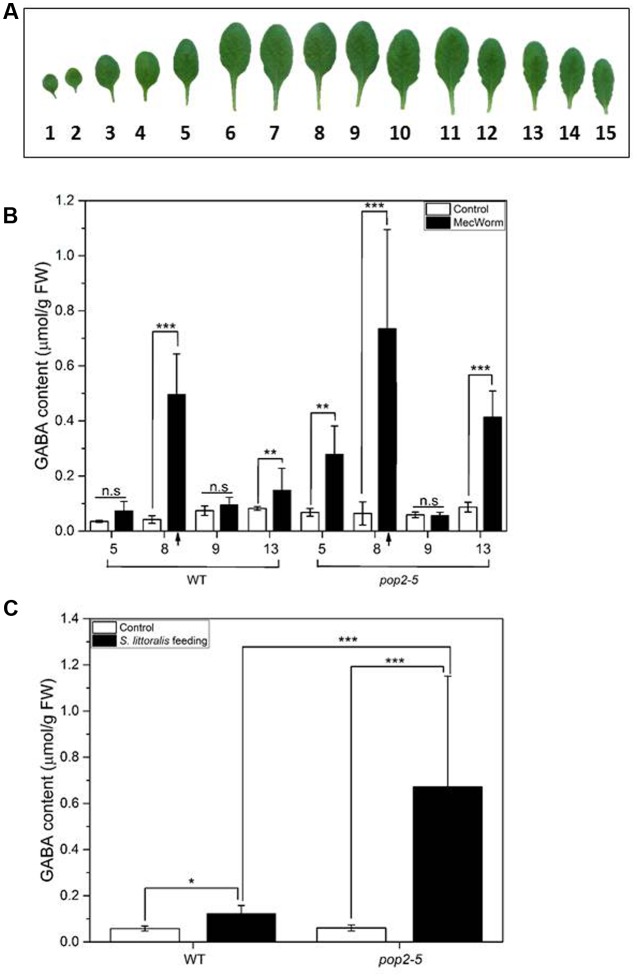
**Accumulation of GABA in individual *Arabidopsis* leaves after MecWorm treatment and *Spodoptera littoralis* short-term feeding assay.** **(A)** Leaves of 4-week old *Arabidopsis thaliana* plants in growing order, from the oldest to the youngest. **(B)** Mean (±SE, *n* = 5) levels of GABA were determined in individual leaves of untreated control plants (white) and plants after treatment with MecWorm for 1.5 h (black). In treated plants, leaf 8 was subjected to mechanical damage (indicated by arrow) and systemic leaves 5, 9, and 11, and treated leaf 8 were analyzed for GABA level. **(C)** Mean (±SE, *n* = 5) levels of GABA were determined in individual leaves of untreated control plants (white) and plants after feeding of third instar *S. littoralis* larvae for 1.5 h (black). Significant differences between the GABA level were analyzed by *t*-test (for each leaf separately, *p* < 0.05, Mann–Whitney *U*-test), ^∗^*P* ≤ 0.05; ^∗∗^*P* ≤ 0.01; ^∗∗∗^*P* ≤ 0.001. No significant difference in GABA concentration was found between untreated genotypes.

We further investigated the GABA response upon MecWorm wounding in well-developed leaves of *pop2-5* plants (**Figure 3A**). With MecWorm we are able to investigate the impact of the isolated wounding process without the contribution of insect-derived compounds in oral secretions (Mithöfer et al., 2005). As shown in **Figure 3B**, continuous mechanical wounding of leaf 8 significantly elevated the amount of GABA in the treated leaf in both WT and *pop2-5* plants. For WT plants, apart from the local GABA accumulation, we observed a significant increase of GABA in leaf 13, which is directly connected to leaf 8 and a non-significant increase in the indirectly connected leaf 5 (Dengler, 2006). For *pop2-5* plants the local GABA accumulation of leaf 8 was followed by a significant increase of GABA in leaves 5 and 13. In preliminary experiments we observed that the GABA elevation in the adjacent leaves is a time-dependent response, and after 90 min of feeding-like wounding of leaf 8 a significant increase of GABA was detectable in leaf 13. As expected, there was no significant increase of GABA on leaf 9, as it is not connected to treated leaf 8. Analysis of the GABA concentration after 1.5 h of *S. littoralis* feeding revealed that the larvae caused a 6-fold higher GABA accumulation in *pop2-5* compared to WT plants, in the fed leaves (**Figure 3C**). The synthesis of GABA in *pop2-5* plants during treatments should be the same but in WT there is more GABA degradation by the transaminase during the assay, so the accumulation at the end was lower in the WT (**Figure 3C**). Here, it should be mentioned that in this set of experiments no clear difference in GABA concentrations between untreated WT and *pop2-*5 plants was found. This contradicts earlier findings including our own study (Palanivelu et al., 2003; Miyashita and Good, 2008; Clark et al., 2009; Scholz et al., 2015) where in *pop2-5* plants a higher GABA level was detected. However, this might be explained by different growth conditions, i.e., long vs. short day. Nevertheless, taken together, the results indicate that also in *pop2-5* mutant plants GABA is an efficient inducible systemic defense factor against insect feeding.

### Jasmonate Levels Are Not Affected in the *pop2-5* Mutant

**FIGURE 4:**
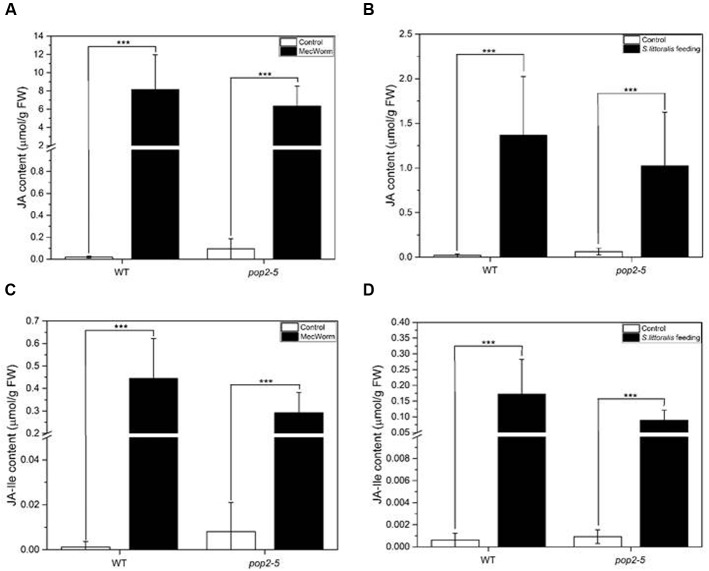
**Accumulation of jasmonates in *Arabidopsis* leaves after *Spodoptera littoralis* short time feeding assay and MecWorm treatment.** JA levels were analyzed after mechanical wounding for 1.5 h **(A)** and after *S. littoralis* feeding for 3 h **(B).** JA-Ile levels were analyzed after mechanical wounding for 1.5 h **(C)** and after *S. littoralis* feeding for 3 h **(D).** For MecWorm treated plants, leaf 8 was subjected to mechanical damage and analyzed for jasmonate level. For quick feeding assay hormone level was analyzed from local fed leaves of third instar *S. littoralis* feeding for 1.5 h. Untreated leaves from untreated plants were used as controls. Statistically significant differences between the JA and JA-Ile levels of the control and treated plant were analyzed by Student’s *t*-test (^∗∗∗^*P* ≤ 0.001). No significant differences in JA and JA-Ile levels were found between the analyzed genotypes.

The accumulation of jasmonates was not influenced by different GABA levels in *gad1/2* and *gad1/2 x pop2-5* mutants. Similarly the accumulation of GABA was not changed in *jar1*, a JA-Ile jasmonate signaling mutant (Scholz et al., 2015). We decided to further test the involvement of jasmonates on GABA accumulation also in the non-GABA-degrading *pop2-5* mutant. Therefore we measured the levels of JA and its bioactive derivative, (+)-7-*iso*-jasmonoyl-*L*-isoleucine (JA-Ile), in *Arabidopsis* WT and in *pop2-5* plant*s* both upon mechanical wounding and herbivore treatment. The concentration of JA and JA-Ile significantly increased after feeding assay with *S. littoralis* and after MecWorm treatment in both tested genotypes (**Figure 4**). In all cases the MecWorm treatment caused higher jasmonate accumulation but the non-treated controls showed no differences between WT and *pop2-5* plants. These data support earlier results suggesting that GABA accumulation is not jasmonate-dependent (Scholz et al., 2015) and *vice versa*. However, we cannot completely rule out that an octadecanoic dependent, but JA/JA-Ile independent, signaling pathway might still be involved in GABA accumulation.

### D_2_-GABA Treatment Increases GABA Concentration in Systemic Adjacent Leaves

**FIGURE 5:**
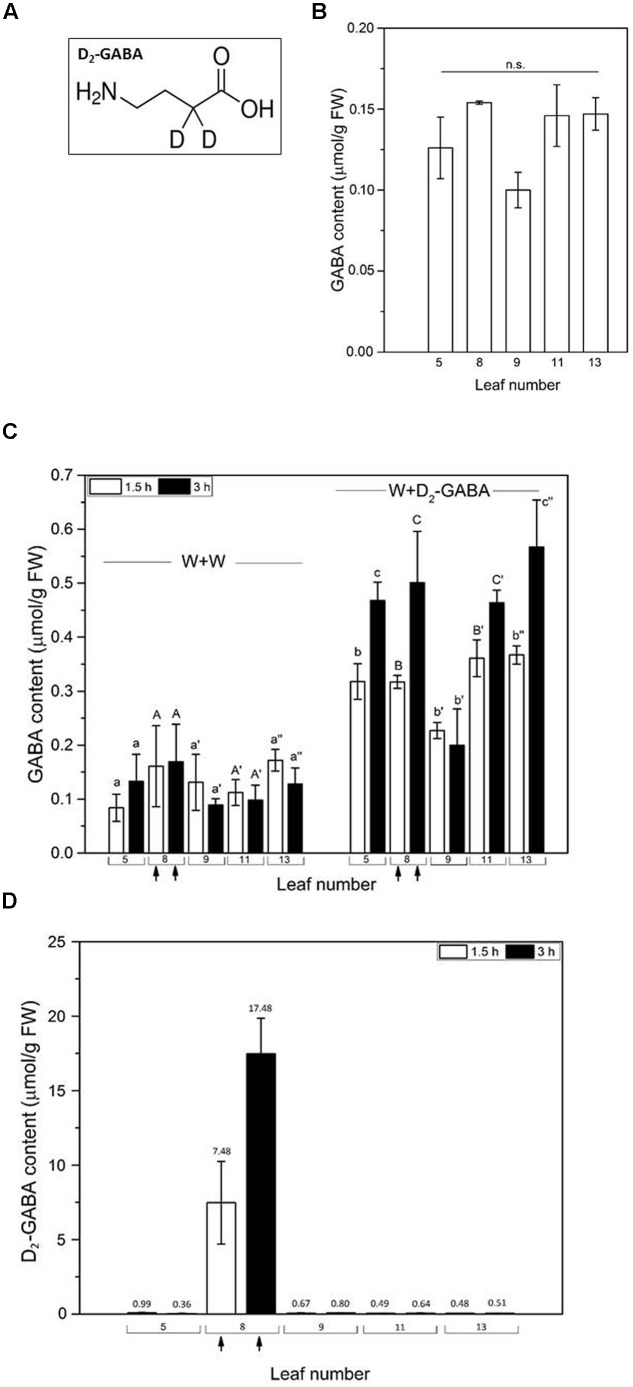
**Accumulation of GABA after isotopically labeled GABA (D_2_-GABA) treatment in *pop2-5* plants.** **(A)** Chemical structure of D_2_-GABA used. **(B)** Mean (±SE, *n* = 7) level of unlabeled GABA in untreated leaves from control plants. **(C)** Mean (±SE, *n* = 7) level of unlabeled GABA in *pop2-5* plants was determined after wounding leaf 8 with a pattern wheel (indicated by arrow), applying water (wounding + water, W + W) or D_2_-GABA (wounding + D_2_-GABA, W + D_2_-GABA) on the wounds and incubating for 1.5 h (white) and 3 h (black). No statistically significant difference was found between the controls (in B) and the wounding plus water treatment. **(D)** Mean (±SE, *n* = 7) levels of D_2_-GABA were determined in different leaves after wounding leaf 8 with a pattern wheel (indicated by arrow), applying D_2_-GABA on the wounds and incubating for 1.5 h (white) and 3 h (black). Values below 1 μmol (g FW)^-1^ do not represent D_2_-GABA but an unknown endogenous compound and should be considered as noise. Significant differences between the treatments were analyzed by One-way ANOVA and Two-way ANOVA (*p* < 0.05, SNK-test) and are indicated by different letters.

To further investigate if GABA is transported to or synthetized in the adjacent leaves, we treated *Arabidopsis pop2-5* plants with the double-deuterated D_2_-GABA (**Figure 5A**) on a leaf that was wounded with a pattern wheel. This approach would enable us to observe any translocation of GABA in the plant leaves, because no D_2_-GABA degradation takes place in the *pop2-5* mutant. The basic GABA levels in different leaves of control *pop2-5* plants were comparable (**Figure 5B**). Leaves of the *pop2-5* plants that were slightly pattern wheel-wounded and incubated with water, showed a GABA concentration similar to control plants (**Figures 5B,C**), indicating that the weak single mechanical wounding had no effect compared to continuous mechanical wounding achieved with MecWorm treatment. Interestingly, when GABA was measured in leaf 8-wounded *pop2-5* plants that were treated with D_2_-GABA, an increase of non-labeled GABA was detected, with the accumulation of GABA in directly connected (leaf 13) and indirectly connected (leaves 5 and 11) leaves. GABA accumulation was significantly higher after 1.5 h and increased more after 3 h (**Figure 5C**). Even leaf 9 showed, compared to wounding and water treatment (W + W), a slight elevation of unlabeled GABA after the application of D_2_-GABA (W + D_2_-GABA). These results suggest that GABA synthesis was stimulated by the combination of the pattern wheel wounding and D_2_-GABA incubation in both the treated leaf and systemic leaves (**Figure 5C**). When we further analyzed the concentration of D_2_-GABA on *pop2-5* treated plants it was only possible to detect higher D_2_-GABA level in the locally treated leaf 8 indicating that D_2_-GABA was taken up. However, the fact that in the adjacent leaves D_2_-GABA was detected not even nearly as in leaf 8 (**Figure 5D**) suggests that it is not transported from the local wounded leaf to the systemic leaves. This result, combined with the observation that also the systemic cytosolic calcium elevation after wounding is not responsible for activation of GABA synthesis in systemic unwounded leaves, demonstrates that there has to be a different signal initiating GABA synthesis and accumulation in systemic leaves. Previous studies in animals presented data concerning GABA-mediated positive feedback loop (Kamermans and Werblin, 1992; Fenalti et al., 2007) and also in plants a several-fold endogenous accumulation of GABA after external application of GABA has been found for different tissues (Shi et al., 2010; Shang et al., 2011; Malekzadeh et al., 2014; Vijayakumari and Puthur, 2016). Further studies are required to establish a role for phloem B-type cells in long-distance signaling in plants (Shelp, 2012). In a study concerning drought stress tolerance with a Poaceae species, *Agrostis stolonifera*, GABA was shown to enhance the accumulation of GABA and of other amino acids (glycine, valine, proline, 5-oxoproline, serine, threonine, aspartic acid and glutamic acid) (Li et al., 2017). Interestingly, in barley plants it was shown that external application of GABA was able to induce action potentials (APs) accompanied by cytoplasmatic acidification short of the position of the stimulus (Felle and Zimmermann, 2007). APs and other electrophysiological reactions represent conceivable signaling candidates for systemic leaves because they have been shown to be involved in wound-induced leaf-to-leaf signaling (Zimmermann et al., 2009, 2016; Mousavi et al., 2013).

## Conclusion

Wounding of plant tissue and cell disruption caused by feeding insects demonstrated that GABA synthesis and accumulation can be a rapid defense response against invertebrate pests. Other studies proposed that GABA might have a role as signaling molecule, activated upon abiotic stresses. Our results demonstrate that under stress-conditions GABA cannot only act as a defense metabolite, but also as a signaling molecule. The approach with isotopically labeled GABA demonstrates in addition that a high local concentration of GABA in the challenged leaf can trigger a *de novo* synthesis of GABA in systemic untreated leaves. This systemic accumulation of GABA is neither dependent on the systemic cytosolic calcium elevation induced by the wounding nor on the direct transport of GABA from wounded to systemic leaves. The signal responsible for this observation remains still unclear but electrophysiological reactions might represent conceivable signaling candidates.

## Author Contributions

SS, JM, and AM conceived and designed the research; SS, JM, MR, and MH performed the experiments; SS, JM, FL, and AM wrote the manuscript. All authors contributed to the manuscript.

## Conflict of Interest Statement

The authors declare that the research was conducted in the absence of any commercial or financial relationships that could be construed as a potential conflict of interest.
